# B chromosomes are associated with redistribution of genetic recombination towards lower recombination chromosomal regions in perennial ryegrass

**DOI:** 10.1093/jxb/ery052

**Published:** 2018-03-21

**Authors:** John Harper, Dylan Phillips, Ann Thomas, Dagmara Gasior, Caron Evans, Wayne Powell, Julie King, Ian King, Glyn Jenkins, Ian Armstead

**Affiliations:** 1 Institute of Biological, Environmental and Rural Sciences, Aberystwyth University, Aberystwyth, UK; 2 Scotland’s Rural College, Edinburgh, UK; 3 School of Biosciences, University of Nottingham, Sutton Bonington, UK

**Keywords:** B chromosomes, chiasma, genetic mapping, *Lolium perenne*, meiosis, perennial ryegrass, recombination

## Abstract

Supernumerary ‘B’ chromosomes are non-essential components of the genome present in a range of plant and animal species—including many grasses. Within diploid and polyploid ryegrass and fescue species, including the forage grass perennial ryegrass (*Lolium perenne* L.), the presence of B chromosomes has been reported as influencing both chromosome pairing and chiasma frequencies. In this study, the effects of the presence/absence of B chromosomes on genetic recombination has been investigated through generating DArT (Diversity Arrays Technology) marker genetic maps for six perennial ryegrass diploid populations, the pollen parents of which contained either two B or zero B chromosomes. Through genetic and cytological analyses of these progeny and their parents, we have identified that, while overall cytological estimates of chiasma frequencies were significantly lower in pollen mother cells with two B chromosomes as compared with zero B chromosomes, the recombination frequencies within some marker intervals were actually increased, particularly for marker intervals in lower recombination regions of chromosomes, namely pericentromeric regions. Thus, in perennial ryegrass, the presence of two B chromosomes redistributed patterns of meiotic recombination in pollen mother cells in ways which could increase the range of allelic variation available to plant breeders.

## Teaser

Genetic recombination in perennial ryegrass is redistributed in favour of lower recombination regions of chromosomes in the presence of supernumerary B chromosomes. This has potential applications in plant breeding.

## Introduction

B chromosomes are small supernumerary chromosomes which are found in a wide variety of both animal and plant species. While they are reported as having a diverse range of potential phenotypic effects on individual fitness and reproductive biology, they are non-essential to the normal functioning of the organism. B chromosomes do not recombine with the ‘normal’ autosomal (A) chromosome complement and can show non-Mendelian inheritance. This can result in lineage-specific increases in the numbers of B chromosomes per cell, within limits imposed by negative effects on fertility. In plant species, B chromosomes have been studied both for their direct effects on the biology of ‘host’ plants and in order to understand their evolutionary *raisons d’être*—and the link between these two. Historically, there have been some reports that the presence of (variable numbers of) B chromosomes can influence plant vigour and fecundity (Teoh *et al.*, 1976; Jones and Rees, 1982; Holmes and Bougourd, 1989, 1991; Jimenez *et al.*, 1994), however the majority of experimental work has focused on the effects of B chromosomes on different aspects of chromosome biology (reviewed by Jones and Rees, 1982; Jones, 1995; Jones and Houben, 2003; Marschner *et al.*, 2007; Jones *et al.*, 2008; Jones, 2012; Houben *et al.*, 2014; Jang *et al.*, 2016; Ruban *et al.*, 2014) and, latterly, the genomics and transcriptomics of B chromosomes (Carchilan *et al.*, 2009; Pereira *et al.*, 2009; Banaei-Moghaddam *et al.*, 2012; Martis *et al.*, 2012; Kwasniewska and Mikolajczyk, 2014; Huang *et al.*, 2016; Jang *et al.*, 2016; Ma *et al.*, 2017).

The effects of B chromosomes on A chromosome recombination at meiosis has been reported for many different species, focusing on chiasma frequencies, and homologous and homoeologous chromosome pairing control in diploid and polyploid interspecies hybrids. There is no one conclusion as to the effects of B chromosomes on chiasma frequencies, with reports of increases, decreases, and neutral associations across a range of species (see reviews above) and the likelihood of specific A/B genome interactions influencing observed effects (Ortiz *et al.*, 1996; Kousaka and Endo, 2012). In the case of ryegrasses and fescues, the presence of B chromosomes in diploid *Lolium perenne* and *Festuca pratensis* (both 2*n*=2*x*=14) has been associated with overall decreases in chiasma frequencies (Cameron and Rees, 1967; Kopecký *et al.*, 2009*b*). However, in autotetraploid *L. perenne* (2*n*=4*x*=28) and in allohexaploid tall fescue (*Festuca arundinacea*, 2*n*=*6x*=42), opposite effects have been observed, with generally higher chiasma frequencies associated with increasing numbers of B chromosomes (Macefield and Evans, 1976; Jauhar and Crane, 1990). The effect of B chromosomes on chromosome pairing control in hybrids within the Lolium/Festuca grasses seems to be clearer, with the presence of B chromosomes generally associated with a reduction in homoeologous pairing in both diploid and polyploid hybrids (Evans and Macefield, 1972, 1973; Bowman and Thomas, 1973; Jimenez and Jenkins, 1995). This is in agreement with the findings across a range of plant hybrids (reviewed by Jenkins and Jones, 2004).

A common observation within angiosperms is that the distribution of recombination along (A) chromosomes is uneven. For example, in members of the Triticeae, the majority of recombination events occur towards the ends (distal regions) of the chromosome arms (Rees and Dale, 1974; Karp and Jones, 1983; Anderson *et al.*, 2003; Kim *et al.*, 2005; Higgins *et al.*, 2014). As a consequence, the physical positions, among other factors, of genes along chromosomes influence the likelihoods with which they will recombine with other genes on the same chromosome to form new allelic combinations. Thus, this uneven distribution of recombination limits the rate at which plant breeding can generate new phenotypic variation for the selection of improved crop plant varieties. So, while the mechanisms that control and influence meiotic recombination in plants are of fundamental biological interest and, particularly in *Arabidopsis thaliana*, considerable progress has been made in elucidating the underlying genetic pathways (reviewed by Choi and Henderson, 2015; Mercier *et al.*, 2015), there has also been considerable interest among crop plant scientists and breeders as to whether it is possible to influence the frequency and relative position of recombination events along chromosomes (Martinez-Perez and Moore, 2008; Phillips *et al.*, 2012, 2015; Crismani *et al.*, 2013; Moore, 2014; Suay *et al.*, 2014).

Perennial ryegrass is the most widely sown forage and amenity grass species in temperate regions of the world, and is the subject of an intensive plant breeding effort. As with other crop species, limits on chromosome recombination frequencies and positions are factors that can slow the rate of improvement of perennial ryegrass as an agricultural species. Over the last few years, it has become possible to measure recombination directly through the generation of high-density genetic maps using different methods for marker generation, including DArT (Diversity Arrays Technology) markers (Wittenberg *et al.*, 2005). Through the availability of this technology for *L. perenne* (Kopecký *et al.*, 2009*a*; Tomaszewski *et al.*, 2012; King *et al.*, 2013), it has become possible to revisit the conclusions of earlier researchers and to explore at higher resolution associations between the presence of B chromosomes and A genome recombination. In this context, the aims of this study were to: (i) generate *L. perenne* genetic mapping populations using pollen parents containing either two or zero B chromosomes; (ii) compare genetic marker recombination frequencies generated through meioses that occurred in the presence or absence of these B chromosomes; (iii) integrate molecular genetic and cytogenetic studies on measured genetic recombination and observed chiasma frequencies; and (iv) assess the potential use of B chromosomes for modifying meiotic recombination in an important crop plant species.

## Materials and methods

### Plant populations

**Fig. 1.**
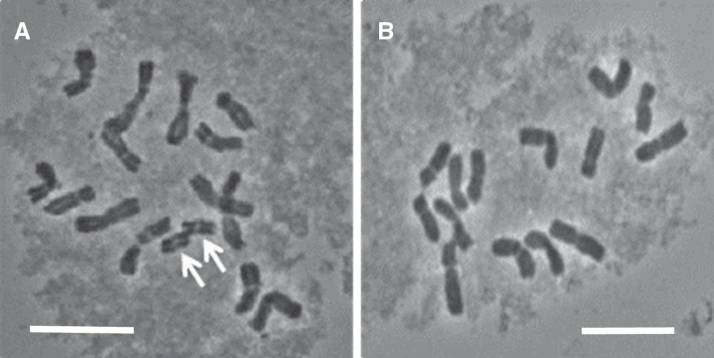
Metaphase chromosome spreads of *L. perenne* 2*n*=2*x*=14 genotypes with (a) and without (b) 2B chromosomes (indicated by white arrows). White bar = 10 µM.

**Fig. 2.**
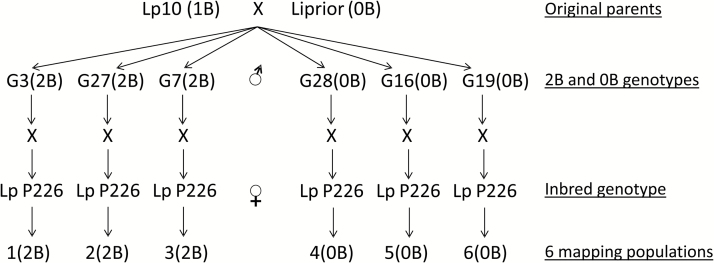
Crossing scheme used to develop the three 0B and three 2B *L. perenne* mapping populations.

The six experimental populations were derived as follows: a single *L. perenne* genotype (Lp10) previously characterized as containing a single B chromosome was pair-crossed as the pollen parent with a single genotype of *L. perenne* from the cultivar Liprior, with zero B (0B) chromosomes, as the female parent. A sample of progeny was characterized cytologically for the presence of B chromosomes (see Fig. 1 for an illustration), and three genotypes with two B (2B) chromosomes and three genotypes with 0B chromosomes were selected. These six genotypes were used for the cytological investigations of chiasma frequencies as described below. Each of these six genotypes was then crossed as the male parent with an *L. perenne* inbred, homozygous line (P226/135/16). The progeny of this second cross derived the experimental populations designated 1(2B), 2(2B), 3(2B), 4(0B), 5(0B), and 6(0B) which were used for the mapping studies (Fig. 2).

### Genotyping data

For the DArT markers, DNA was extracted from 94 genotypes of each of the six genetic mapping families using the QIAGEN DNeasy 96 Plant Kit (QIAGEN, Crawley, UK). These samples were processed on the high-density Lolium/Festuca DArT arrays at Diversity Arrays Technology Pty Ltd, Canberra, Australia and segregating markers were identified. Additionally, the six families were screened with 48 simple sequence repeat (SSR) markers with known genetic map positions in *L. perenne* in order to anchor the DArT maps to existing linkage groups (LGs).

### DArT marker selection

The DArT marker array assayed each genotype for 4519 potential markers. DArT markers show presence/absence-type segregation which would be expected to conform to a 1:1 (BC1-type) ratio in the absence of distorted segregations. Only markers which were present in between 30% and 70% of the genotypes within at least one of the six families and/or had been successfully assigned to LGs of either *L. perenne* or *L. multiflorum* in previous studies (Bartoš *et al.*, 2011; Tomaszewski *et al.*, 2012; King *et al.*, 2013) were used in subsequent steps.

### Genetic map construction

#### Within family

For each of the six families (92–94 genotypes), separate genetic maps were constructed using the Joinmap4.1 (Kyazma, The Netherlands) regression mapping option, the Kosambi mapping function, population type BC1, and using DArT marker genotyping data and a limited number of SSRs to assign LG designations. Mapping was commenced using a LOD threshold of 1.0, recombination frequency (RF) threshold of 0.4, and a jump threshold of 5.0. Final maps consisted of marker orders and distances generated using rounds 1 and 2 of the mapping procedure—excluding markers that exceeded the jump (probability) threshold for inclusion.

#### Consensus maps

For each of the seven LGs, JoinMap4.1 was used to generate a consensus map using the Combine Groups For Map Integration option with mapping parameters as above. For each consensus LG, a ‘starting’ marker order was used (imposing an initial approximate marker order greatly speeds up the mapping process for densely populated LGs). The starting order was determined by the average genetic distance of each marker across the individual family LGs, or just the single map position for markers only present in one family.

#### Comparison of map lengths from equivalent consecutive marker interval (ECMI) markers

**Fig. 3.**
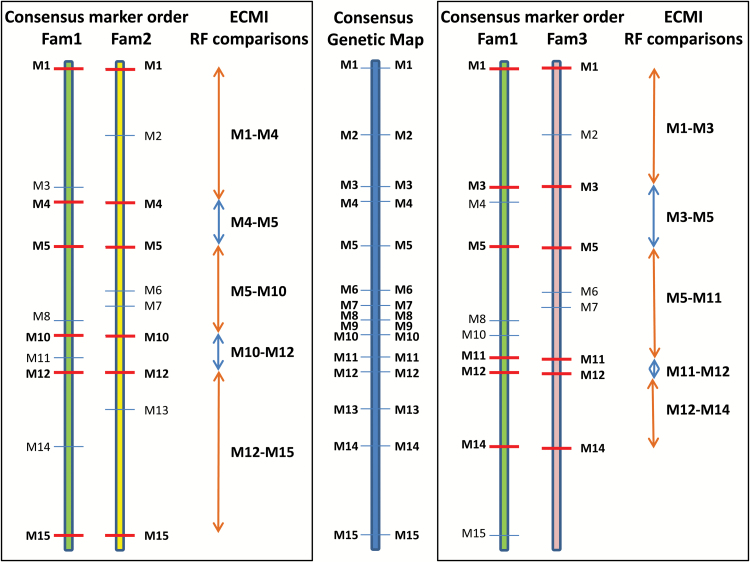
Equivalent consecutive marker intervals (ECMIs). For each linkage group, a consensus genetic map is developed which is used to define the marker order for that linkage group. A subset of the markers present in the consensus map are also present in each of the six families. Markers that are common to any two families (horizontal red bar) define the ECMIs for that family pair. For each ECMI, the recombination frequency (RF) for that ECMI marker pair from each of the two families can be compared. In the example above, M1–M4 RF can be compared between Fam1 and Fam2; the M1–M3 RF can be compared between Fam1 and Fam3.

For each 2B and 0B family comparison, genetic map lengths were generated using only the markers which defined ECMIs (see Fig. 3 and its legend for an ECMI definition) for that family comparison. Maps were constructed as described above except using a LOD threshold of 0 and an RF threshold of 0.5.

### Comparison of recombination frequencies between 2B and 0B families

The consensus maps were used to define a ‘standard’ marker order for each LG which could be applied across all of the six families. This allowed for the identification of ECMIs for each LG comparison between families—intended to reduce the likelihood of comparing overlapping, and so non-independent, intervals. For each of the nine possible pairwise family comparisons, RFs for ECMIs between all combinations of 2B and 0B families were extracted. For ECMIs, the RF calculated from the 0B family was subtracted from the RF calculated from the 2B family to derive a positive, negative, or zero score for that ECMI. The results were analysed family-wise and LG-wise using a two sample sign test excluding zero scores. Additionally, ECMIs between 2B and 0B families were analysed for significant differences using the χ^2^ module in JoinMap4.1. A 10% Benjamini–Hochberg false discovery rate (BH-FDR; Benjamini and Hochberg, 1995) was used to control for multiple testing.

### Cytological estimation of chiasma frequencies and positions

Emerging inflorescences were taken from the six pollen parents used in producing the six mapping populations: three pollen parents with 0B and three with 2B chromosomes. The inflorescences were taken as they emerged from the flag leaf and were fixed in Carnoy’s fixative (6:3:1 ethanol/chloroform/acetic acid) for 24 h. They were then removed and placed in 70% alcohol for a further 24 h, and finally stored in 70% alcohol at 4 °C until analysed.The heads were removed from alcohol and squashes made in acetocarmine stain to find cells at metaphase I. Subsequent analysis of chiasmata used a Leica DM/RB epifluorescence microscope. The frequencies of chiasmata were scored in 100 pollen mother cells (PMCs) in each of the plants. For estimation of chiasma positions, 350 rod bivalents for each family were scored as either distal, interstitial, or proximal after the method of Karp and Jones (1983).

## Results

### Genetic mapping

Using DArT and SSR markers mapped to identified LGs in previous studies as a guide for LG designation (Bartoš *et al.*, 2011; Tomaszewski *et al.*, 2012; King *et al.*, 2013), a total of 1322 DArT and 13 SSR markers were assigned to LGs across the six families and the consensus maps (Table 1; Supplementary Table S1 at *JXB* online). Twenty-eight DArT markers and one SSR marker were assigned to one of two different LGs in different families, indicating that these markers can potentially detect more than one locus. This gave a total of 1360 loci across the seven consensus LGs. Only 13% of markers were assigned to LGs across at least four of the six families, and only 1% could be assigned to all six, limiting the number of common marker intervals that could be compared across all of the 2B and 0B families (Table 2). Across the LGs, an average of 31% of the markers were present in only one of the families.

**Table 1.** Number of markers and centiMorgan length of linkage groups across the six families and within the consensus maps

		LG1		LG2		LG3		LG4		LG5		LG6		LG7		Total	
Family	2B/0B^*a*^	No.	cM	No.	cM	No.	cM	No.	cM	No.	cM	No.	cM	No.	cM	No.	cM
**1(2B**)	2B	48	**74**	48	**98**	48	**88**	87	**134**	39	**97**	96	**97**	74	**93**	437	**662**
**2(2B**)	2B	63	**87**	39	**89**	73	**78**	123	**129**	91	**60**	30	**65**	80	**99**	499	**607**
**3(2B**)	2B	47	**72**	58	**84**	79	**107**	91	**164**	63	**63**	86	**105**	79	**134**	529	**729**
**4(0B**)	0B	54	**77**	47	**88**	89	**113**	123	**121**	76	**45**	113	**92**	83	**117**	585	**653**
**5(0B**)	0B	85	**84**	55	**78**	70	**90**	67	**151**	76	**58**	39	**87**	117	**92**	509	**640**
**6(0B**)	0B	42	**85**	44	**94**	73	**110**	107	**108**	51	**50**	108	**84**	90	**53**	515	**584**
**Consensus**	–	138	**88**	105	**93**	214	**94**	276	**122**	183	**90**	197	**113**	247	**91**	1360	**691**

^*a*^ Pollen parent in the cross from which the mapping family was derived containing two (2B) or zero (0B) B chromosomes.

**Table 2.** The frequency (%) with which markers assigned to particular linkage groups (LGs) were mapped across the six possible families

No. of families	% of markers assigned to LGs							
	LG1	LG2	LG3	LG4	LG5	LG6	LG7	Overall
**1**	29	21	47	31	30	24	32	31
**2**	30	15	25	38	34	35	41	33
**3**	21	39	17	18	27	28	16	22
**4**	10	17	6	10	8	7	9	9
**5**	7	6	3	2	1	3	2	3
**6**	3	2	2	1	0	3	0	1

A number of markers on the individual LGs showed quite considerable segregation distortions away from the 1:1 ratio particularly for the LGs relating to chromosomes 1, 2, 5, and 6. Overall, ~35% and 48% of the markers scored showed significantly distorted segregations (*P*<0.05) for the 2B and 0B families, respectively.

### Comparison of recombination frequencies between 2B and 0B populations

As a consequence of the few markers that were mapped in common across the six families, the analysis focused on individual 2B versus 0B ECMI comparisons on a family by family basis. This allowed for a total 1658 pairs of RFs to be compared from ECMIs between 2B and 0B families (Table 3). Overall, among the 1658 ECMI RF comparisons, in ~50% of the cases the RF derived from the 2B family was greater than the RF derived from the 0B family, with the reverse being the case ~28% of the time; ~22% of the RF comparisons showed no difference in RFs. This represents a highly significant excess (~1.8:1) of positive scores and indicates a trend for larger RFs for ECMIs in the 2B families (Table 3A). This trend was also observed on the LG by LG basis (Table 3B) and the family by family basis (Table 3C) for the seven out of nine individual family comparisons which were significant (*P*<0.05).

**Table 3.** Numbers of positive, negative, and zero differences between RFs of equivalent consecutive marker intervals for the 2B versus 0B family and LG comparisons

Comparison	Total and individual numbers of differences between RFs per comparison				Two-sample sign test	
	Total	Positive	Negative	Zero	*P*-value	
(A) Overall RF comparison between 2B and 0B families^*a*^						
All	1658	831	460	367	<0.001	***
(B) RFs compared by LG^*a*^						
LG1	232	128	69	35	<0.001	***
LG2	210	117	63	30	<0.001	***
LG3	234	111	70	53	0.003	**
LG4	277	139	84	54	<0.001	***
LG5	206	81	52	73	0.015	*
LG6	279	138	77	64	<0.001	***
LG7	220	117	45	58	<0.001	***
(C) RFs compared between individual 2B and 0B families^*a*^						
1(2B) versus 4(0B)	186	81	59	46	0.09	NS
1(2B) versus 5(0B)	121	66	39	16	0.014	**
1(2B) versus 6(0B)	183	103	38	42	<0.001	***
2(2B) versus 4(0B)	199	73	76	50	0.87	NS
2(2B) versus 5(0B)	170	81	48	41	0.005	**
2(2B) versus 6(0B)	124	61	40	23	0.046	*
3(2B) versus 4(0B)	215	116	48	51	<0.001	***
3(2B) versus 5(0B)	257	130	72	55	<0.001	***
3(2B) versus 6(0B)	203	120	40	43	<0.001	***

^*a*^ For each comparable interval, the 0B family RF is subtracted from the 2B family RF to derive the difference.

ECMI RF differences were also analysed for significance using the χ^2^ module in JoinMap4.1. From the total number of 1658 ECMI comparisons, this identified 221 comparisons with *P*≤0.05, of which 54 remained below the 10% BH-FDR threshold (excluding ECMIs where the RF=0 in both 2B and 0B families). ECMI RF differences for 2B versus 0B families were also disproportionally positive for the entire 221 χ^2^ significant ECMIs (163 positive/58 negative). For the 54 χ^2^ significant ECMIs that remained below the 10% BH-FDR threshold, 42 were positive and 12 were negative.

### Recombination frequencies and map distances

Table 1 gives the overall map distances assembled for each of the six families, and the average total map distances for the 2B and 0B families were 666 cM and 625 cM, respectively. To derive comparisons of map distances defined by common markers, maps were also generated for each 2B versus 0B comparison using just the markers which defined the ECMIs for that comparison. Of these nine possible comparisons across all of the LGs, eight described larger centiMorgan distances in the 2B family as compared with the 0B family (Table 4). On average, this suggested an ~10% greater recombination distance overall in the families derived from male parents containing 2B chromosomes.

**Table 4.** Comparative map lengths using just the markers which define the ECMIs for each of the nine possible 2B and 0B family comparisons

		2B/0B family comparison									Total
**2B** ^***a***^	**family**	1(2B)	1(2B)	1(2B)	2(2B)	2(2B)	2(2B)	3(2B)	3(2B)	3(2B)	
	**cM**	602	537	546	448	482	414	563	644	490	4726
**0B** ^***a***^	**family**	4(0B)	5(0B)	6(0B)	4(0B)	5(0B)	6(0B)	4(0B)	5(0B)	6(0B)	
	**cM**	544	511	436	471	465	365	532	510	418	4252
**2B/0B % difference**		10	5	25	-5	4	13	6	26	17	11

^*a*^ Differences between 2B and 0B family maps *P*<0.05 (paired sample *t*-test).

### Cytological estimation of chiasma frequencies and distributions

**Fig. 4.**
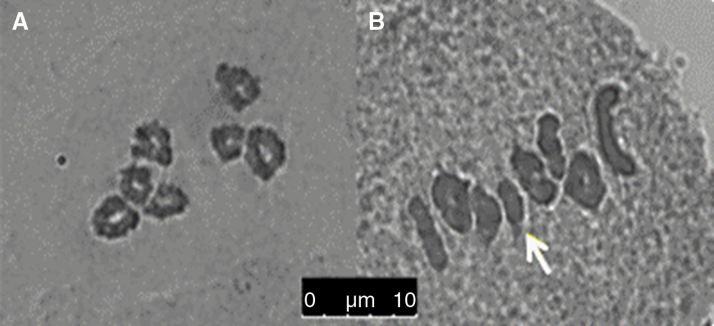
Pollen mother cell chromosome spreads of *L. perenne* 2*n*=2*x*=14 genotypes at metaphase1. (a) Genotype with no B chromosomes pairing as seven ring bivalent configurations; (b) genotype with four ring and three rod bivalent configurations, and two B chromosomes pairing as a ring arrowed in white.

One hundred PMCs from each of the male parental genotypes of the crosses were analysed using light microscopy to estimate chiasma frequencies (Table 5; see Fig. 4 for examples) directly from chromosome spreads. According to these observations, the average chiasma frequency per cell in the 2B families was 10.9; in the 0B families, this frequency was 12.1 (*P*<0.001). This observation suggests that the recombination rate in the male parent is reduced by the presence of 2B chromosomes—the opposite result to that implied by the molecular marker analysis. The major effect of the 2B chromosomes was not in notably increasing or reducing the rate of ‘aberrant’ chromosome associations (i.e. quadrivalent, trivalent, or univalent), although the total number of univalents observed in the 2B families was larger than that in the 0B families (32 versus 15, respectively). The main influence of the presence of 2B chromosomes was instead on the number of ring (chromosome pairs joined through one or more chiasmata in each arm) versus rod (chromosome pairs joined through one or more chiasmata in one arm only) bivalent associations, with the overall ratio of rings to rods in the 2B and 0B families being 1:1 and 2:1, respectively.

**Table 5.** Chiasma counts from 100 pollen mother cells in the male parental genotype for each cross

Family	Pollen mother cells	Total chiasmata	Chiasmata per cell ^*a*^	Quadri- valents	Trivalents	Univalents	Bi valents	Ring bivalents	Rod bivalents	Ratio rings to rods
**1(2B**)	100	1097	11	2	0	4	694	346	348	0.99
**2(2B**)	100	1086	10.9	1	2	12	689	372	317	1.17
**3(2B**)	100	1093	10.9	0	0	8	696	365	331	1.1
**$\bar{\text{X}}$**	100	1076	10.9	1	0.7	8	693	361	332	1
**4(0B**)	100	1202	12	0	0	2	699	467	232	2
**5(0B**)	100	1160	11.6	0	1	7	695	420	275	1.5
**6(0B**)	100	1253	12.5	0	1	5	696	490	206	2.4
**$\bar{\text{X}}$**	100	1205	12.1	0	0.7	4.7	696.7	459	237	2

^*a*^ Differences between the 2B and 0B families are significant at *P*<0.05.

## Discussion

### Genetic mapping and ECMIs

#### DArT marker frequencies

King *et al.* (2013) compared two different *L. perenne* genetic maps based on DArT markers, and identified that ~28% were in common between the two populations. In the present study, the average number of DArT markers shared between any two of the families was ~33% (Table 2). Thus, the number of shared markers across the different families on a pair-wise basis is consistent with this previous study. Ideally, there would be many DArT markers mapped in common across all six families as this would have allowed for multiple comparisons between 2B and 0B families of RFs for the same marker intervals. However, as Table 2 illustrates, the number of DArT markers mapped in common across all six families was ~1% of the total. Thus, there does not seem to be a ‘core set’ of DArT markers consistently present across different *L. perenne* mapping families, and DArT marker presence/absence is relatively family specific.

Many of the markers scored showed significantly distorted segregations though there was no obvious association with the presence or absence of B chromosomes. *Lolium perenne* has been identified as containing a two locus gametophytic self-incompatibility (SI) system which militates against inbreeding but can also influence cross-compatibilities more widely. This is controlled, primarily, by the *S* and *Z* loci on chromosomes 1 and 2, respectively, though it has been established that there are a number of other loci which can also influence self-fertility, for example also on chromosomes 3, 5, and 6 (Thorogood *et al.*, 2017). The patterns of segregation distortions observed in this experiment are not inconsistent with a genetically mediated incompatibility response—though it is beyond the scope of this study to investigate this further.

#### Consensus maps

A consequence of using large and unevenly distributed (in terms of genetic distance) markers sets and relatively small population sizes (92–94 genotypes) is that there will be uncertainty in terms of the ‘correct’ marker order (i.e. the actual linear order of marker sequences along the chromosomes relating to the LGs). In order to define a consistent marker order which could be applied across the marker sets developed for each family, consensus maps were derived as described in the Materials and methods and Supplementary Table S1. The consensus maps are, effectively, an ‘average’ of the marker orders predicted for the individual families and so are only an estimate of the true order. Inaccurate consensus marker orders may mean that some consecutive ECMIs on an LG overlap to a degree. While this is a potential source of error relating to the independence of the RF differences associated with consecutive ECMIs, it does not introduce a systematic bias in terms of recovering more or less positive or negative estimates for 2B versus 0B ECMI RF comparisons.

#### Potential influence of genotyping errors

Genotyping errors can be indicated by the presence of ‘singleton’ marker scores (apparent double recombinations around a single marker), though a singleton marker is not necessarily a genotyping error. The effect of such genotyping errors is to inflate estimates of recombination in the region of the singleton, which can lead to genetic map expansion. In this study, the frequency of singletons was ~1% across both 2B and 0B families and, so, any map expansion due to error would have affected estimates of recombination approximately evenly for 2B and 0B families. The families are illustrated as graphical genotypes in Supplementary Table S2.

### The extent and position of recombination within linkage groups and chromosomes

**Fig. 5.**
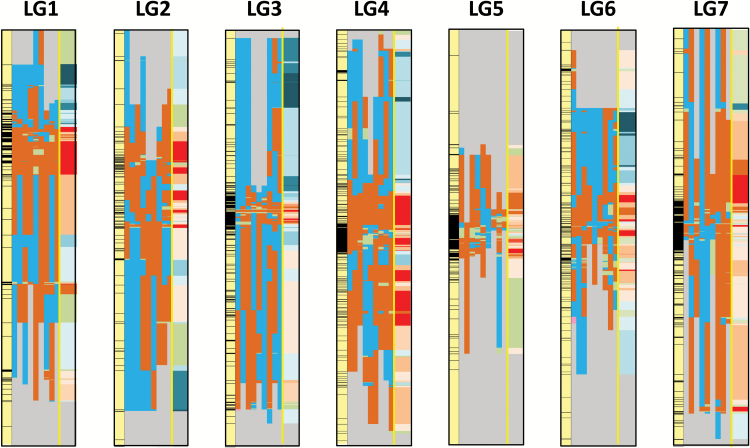
Diagrammatic representation of the distribution along the LGs of positive and negative differences in RFs for ECMIs compared between 2B and 0B families. The first column (yellow) for each LG indicates the marker (black horizontal bars) distribution for the consensus map. For each LG, the nine narrow columns represent the nine possible 2B and 0B family comparisons; from left to right 1(2B)/4(0B), 1(2B)/5(0B), 1(2B)/6(0B), 2(2B)/4(0B), 2(2B)/5(0B), 2(2B)/6(0B), 3(2B)/4(0B), 3(2B)/5(0B), and 3(2B)/6(0B); colour codes indicate whether the 2B minus 0B RF difference for an ECMI was positive (orange), negative (light blue), or zero (light green). Grey indicates a region of an LG not covered within an ECMI. The tenth, wider column, delimited by a yellow vertical line, is the ‘average’ across the nine different family comparisons for each LG; each positive 2B minus 0B RF difference in each family comparison is given a score of +1 and each negative difference a score of –1. The total across the nine possible family comparisons for each interval for each LG is then illustrated as a colour code: light orange to red=increasing numbers of positive differences; light blue to dark blue=increasing numbers of negative differences; green=zero; grey=no ECMIs identified in this region.

Total high-density genetic map lengths reported for *L. perenne* can vary considerably (e.g. Tomaszewski *et al.*, 2012; Hegarty *et al.*, 2013; King *et al.*, 2013; Paina *et al.*, 2016; Velmurugan *et al.*, 2016) depending on the mapping approach used (i.e. mapping function, algorithm, stringency) and the marker type and density. Published studies using methods comparable with those described in this study have identified total map lengths of 683 cM (King *et al.*, 2013) and 506 cM (Hegarty *et al.*, 2013), similar to the 584–730 cM spread identified across the six populations mapped in the present study. Thus, we can assume that marker coverage was in the range expected for this type of analysis. Looking at overall differences between 2B and 0B maps, the total lengths derived from the 2B families were ~10% greater than those derived from the 0B families (Tables 2 and 4)—which might indicate an effect of 2B chromosomes on recombination frequencies across the A genome. However, total map lengths (whichever analysis method is used) are heavily influenced by the uneven distribution of recombination along chromosomes with often relatively larger and variable estimates of centiMorgan distances between the fewer, terminally located markers in LGs of *Lolium* spp. (see Fig. 5 for an illustration of this from the marker distribution within the consensus LGs in this study). This is in accordance with the cytological observation that the majority of chiasmata occur towards the ends of chromosome arms in ryegrasses and many grass species (Rees and Dale, 1974; Karp and Jones, 1983; Higgins *et al.*, 2014). In the present study, instead of focusing on the overall genetic distance (a composite of RFs and mapping function) for the analyses described in Table 3 and Fig. 5, we have given each ECMI RF difference that could be evaluated between 2B and 0B families equal weight; that is, the comparisons made relate to the directions (positive, negative, or zero) of ECMI RF differences rather than their magnitude. On this basis, out of a total of 1658 ECMI RF comparisons between 2B and 0B families, the ECMI RFs derived from the 2B families were larger than those derived from the 0B families approximately twice as often as they were smaller or the same (*P*<0.001; Table 3A). This trend was conserved when the data were analysed on an LG or family basis (Table 3B) or when just considering only individual ECMI RF comparison differences with *P*≤0.05 (see ECMI RF comparisons); in this latter case, the positive:negative ratio of ECMI RF differences was ~3:1. The numbers and distribution of this subset of ECMI RF differences are given and illustrated in Table 6. Noticeably, while the overall distribution of ECMIs between LGs is relatively even (between 10% and 17% per LG), the distribution of the subsets of ECMI RF differences with *P*≤0.05 and within the BH-FDR threshold is less even, with LGs 1 and 4/6 showing the greatest differences. From a number of genetic mapping studies in *L. perenne* (see references above), LG4 is consistently the longest LG, indicating that this chromosome recombines more frequently (or, at least, genetic markers capture more of the recombination for LG4). Similarly, in this study, LG1 has the shortest consensus map and the second shortest average total genetic distance across the six families—though this is a less consistent outcome across previous studies. So, it is possible that 2B chromosomes may have a preferential influence on recombination in particular chromosomes, as has been noted in rye and *Crepis capillaris* (White and Rees, 1985; Parker *et al.*, 1990). However, the B chromosomes may just be acting in such a way as to magnify a pre-existing chromosome-specific trend in observed recombination differences.

**Table 6.** The distribution of individually significant ECMI RF differences between 2B and 0B families according to LG, excluding ECMIs where the RF=0 for both 2B and 0B families

	ECMI RF comparisons					
	Total		*P*≤0.05		Within 10% BH-FDR threshold	
LG	*n*	%	*n*	%	*n*	%
**1**	200	15.4	15	6.8	1	1.9
**2**	182	14.0	19	8.6	3	5.6
**3**	182	14.0	40	18.1	5	9.3
**4**	223	17.1	55	24.9	17	31.5
**5**	134	10.3	15	6.8	5	9.3
**6**	219	16.8	51	23.1	18	33.3
**7**	162	12.4	26	11.8	5	9.3
**Total**	1302		221		54	

The majority of previous studies on the effects of B chromosomes on recombination across a range of plant species have relied on cytogenetic estimations of chiasma frequencies, rather than marker-based genetic mapping, and in many of these studies the presence of B chromosomes is associated with an overall increase in chiasma frequencies (including in autotetraploid *L. perenne* and *F. arundinacea*; Macefield and Evans, 1976; Jauhar and Crane, 1990). In contrast, the results of the present study indicated that the presence of 2B chromosomes was associated with a significant overall reduction in chiasma frequencies, in line with previous results in diploid *L. perenne* and *F. pratensis* (Cameron and Rees, 1967; Kopecký *et al.*, 2009*b*). A pilot study for the experiments reported here, using the same plant material but carried out in a previous year, supported this conclusion (data not shown). Therefore, the influence of the presence of 2B chromosomes in depressing overall chiasma frequencies in these species seems to be a reliable observation. This raises some questions. If we assume that there is a direct concordance between recombination frequencies measured through the use of molecular markers and chiasma frequencies observed using cytological assays (King *et al.*, 2002), we would expect to come to similar conclusions using both methods on the influence of 2B chromosomes on recombination/chiasma frequencies. However, the molecular marker approach indicated that the 2B families generated marginally larger genetic maps than the 0B families (average 667 cM versus 625 cM; Table 1), whereas the cytological data would predict the opposite effect, with average map distances of 545 cM versus 605 cM (estimate based on one chiasma per 50 cM of genetic distance) for the 2B and 0B families, respectively. While these differences in overall map lengths are not substantial (comparisons between data in Table 1 are problematic due to differing marker sets and, so, marker coverage), the significant differences described in Table 3 indicate an effect of the 2B chromosomes in increasing RFs in the direction indicated by the genetic maps; this is substantiated by the significant differences (*P*<0.05) between the ECMI-based genetic map comparisons (which do use common marker sets) described in Table 4. The most likely explanation for any inconsistencies between molecular and cytological approaches is that they are not equivalent in terms of their coverage of the chromosomes. Whereas cytological assays observe the entire chromosome, molecular markers only sample those regions of chromosomes tagged by LGs and, in the case of the RF comparisons in this study, ECMIs. Thus, a proportion of the distal chiasmata may occur between the physical position on a chromosome of the terminal molecular marker in the LG relating to that chromosome and the telomere. However, other factors may contribute; for example, the number of recombination events associated with chromosome configurations scored cytologically as single chiasma may be an underestimate and 2B chromosomes may promote an increased number of recombination events per visible chiasma. Also, gene conversion events detectable by the molecular marker assays may occur at higher frequencies in the presence of 2B chromosomes; these will be ‘invisible’ to the cytological assays. However, in the case of gene conversion, one might also expect to see a higher frequency of singleton marker scores in the mapping data, and this was not evident; the average singleton percentage across the 2B and 0B populations was ~1% in both cases.

It is apparent that due to the lack of common markers between families towards the ends of LGs, ECMI coverage was limited in these regions (Fig. 5). This would imply that, in cytogenetic terminology, we are focusing primarily on interstitial and proximal rather than distal chromosome regions. Following directly from this, the significant RF differences between 2B and 0B families described in Table 3 and illustrated in Fig. 5 indicate that, while overall chiasma frequencies may have been reduced in the 2B families, the distribution of the remaining chiasmata has been shifted from distal to more interstitial and proximal regions relative to the 0B families. We attempted to confirm this cytologically using the scoring methods for distal, interstitial, and proximal chiasma positions described by Karp and Jones (1983) and, while the resolution of this approach is limited, the results did indicate that a greater proportion of chiasmata were found in the interstitial and proximal regions in the 2B families—though with noticeable interfamily variations (Supplementary Table S3; Fig. S1). It is hoped that we will be able to confirm this finding further with greater resolution using chromosome position-specific fluorescent *in situ* hybridization (FISH) tags at some point in the future. Previous studies on the effects of B chromosomes on meiosis in *L. perenne* and related species have not recorded observations on the relative positions of chiasmata within chromosomes, other than rod versus ring configurations, and so it is not established as to whether this is a generic finding. However, in a study looking at the effects of *AA* (*A* and *C* here refer to specific *Brassica* subgenomes) homologous genome recombination in *Brassica* hybrids containing variable numbers of *C* genome univalents, while the presence of the univalents markedly increased the overall genetic map sizes, the distribution of recombination remained more or less unchanged (Suay *et al.*, 2014). So, changes in recombination frequencies do not necessarily imply changes in recombination positions.

### Changing patterns of recombination—potential opportunities for plant breeding

An axiom of plant breeding is that increasing the number of allelic variants within a breeding population will make available a greater range of favourable combinations of alleles which can be identified through specific selection regimes. The outcomes of our study indicate that, for perennial ryegrass, the presence of B chromosomes results in a relatively higher rate of recombination in non-distal, lower recombination regions. Practically speaking, if this could be replicated in a breeding population, this should mean a reduction in the amount of ‘redundant’ (frequently obtained) and an increase in the amount of ‘effective’ (rarely obtained) recombination for the plant breeder—thus leading to an increased rate of germplasm improvement for each unit of time and resources.

B chromosomes as a potential tool for manipulating recombination in plant breeding would have some appeal as they would be relatively easy to introduce into and remove (if required) from a population by standard crossing procedures. In this context, while at present individual genotypes containing B chromosomes are identified cytologically, the application of flow cytometry and/or the development of a molecular diagnostic(s) are likely to be straightforward and are well suited to the type of high-throughput and low-cost analyses that are required by plant breeding programmes. Additionally, while increasing numbers of B chromosomes often impact on fertility and so seed production, there were no noticeable difference between the 0B and 2B parents in terms of either pollen viability or the derived seed set in the common female parent in this experiment (data not shown). This is in contrast to the effects of *C* subgenome univalents in *Brassica* which, while increasing recombination rates, had negative effects on fertility (Suay *et al.*, 2014). Thus, in the case of an outbreeding species such as perennial ryegrass, one could foresee that in earlier rounds of plant breeding selection when it is desirable to maximize the genetic variation, the plant breeder could work with B chromosome-containing material; in subsequent seed multiplication steps where, in an outbreeding crop, the challenge is to limit the amount of new phenotypic variation in order to conform to variety uniformity and stability guidelines, the breeder could identify and multiply only those genotypes without B chromosomes.

B chromosomes would also offer a particular advantage over genetic modifiers of recombination located on the A chromosome complement in that B chromosome-associated effects would remain stable over multiple generations. This is in contrast to polygenic modifiers of recombination on the A genome which are likely to be less predictable, particularly in an outbreeding population, or genes of major effect which, unless fixed throughout the population, can recombine themselves out of the favourable genetic backgrounds. Additionally, in the cases where polygenic or major effect genes were fixed in a population, the resulting enhanced recombination would still be apparent in seed multiplication steps for variety production, possibly leading to problems in terms of variety uniformity.

### Future work

There are two very fruitful areas in which research into the effects of B chromosomes on recombination could be extended.

First, what are the molecular mechanisms by which B chromosomes alter patterns of recombination? It has been observed that rye and maize B chromosomes can contain transcribed genes (Carchilan *et al.*, 2009; Banaei-Moghaddam *et al.*, 2012; Huang *et al.*, 2016; Ma *et al.*, 2017) and multiple genic fragments with homology to A genome gene sequences (Martis *et al.*, 2012). So, it is possible that dosage or interference effects may influence the relative positions of chiasmata by reducing the location specificity. It has also been observed that B chromosomes in rye can influence the distribution of histone methylation—with consequences for the products of meiosis (González-Sánchez *et al.*, 2014). So, whether B chromosomes influence recombination through a direct effect on the mechanisms of the formation of synaptonemal complexes and the loading of recombination machinery or, indirectly, possibly by influencing chromatin condensation and/or the epigenetic profiles of chromosomes, is unknown but of considerable interest.

Secondly, in exploring the potential for exploiting B chromosomes in ryegrass breeding, the important next steps will be to introduce B chromosomes into both elite ryegrass breeding backgrounds and less-developed gene pools which are being targeted for selection, followed by observation of their effects in terms of both molecular recombination and phenotype. If the increased recombination in non-distal areas of the genome does generate new allelic combinations, then these should become apparent in terms of an extended range of phenotypic variation or reduced population sizes required to obtain an existing level of phenotypic variation. More generically, it will be interesting to see if similar effects of B chromosomes in increasing the frequency of recombination in non-distal chromosomal regions can be replicated in other crop species, including cereals.

## Supplementary data

Supplementary data are available at *JXB* online.

Table S1. Consensus genetic maps for LGs 1–7.

Table S2. Graphical genotype representations of mapping family individuals.

Table S3. Chiasma positions scored in 350 bivalents from each family according to the method of Karp and Jones (1983).

Figure S1. Illustration of *L. perenne* chromosome pairing conformations at metaphase.

Supplementary Table S1

Supplementary Table S2

Supplementary Table S3 and Figure S1
