# Supplementary material for: B chromosomes are associated with redistribution of genetic recombination towards lower recombination chromosomal regions in perennial ryegrass
Source: J Exp Bot. 2018 Mar 21;69(8):1861–71. doi: 10.1093/jxb/ery052 (PMC6019035; doi:10.1093/jxb/ery052)
Supplement: Supplementary Table S3 and Figure S1 [file ery052_suppl_supplementary_table_s3-figure_s1.pdf]

Supplementary Table S3. Chiasma positions scored in 350 rod bivalents from each family according to the method of Karp and Jones (1983)

Family	Distal	Chiasma positions<br>Interstitial	Proximal
**1(2B)**	396	126	40
**2(2B)**	485	73	29
**3(2B)**	425	131	33
$\bar{x}$	*435.3*	*110*	*34*
**4(0B)**	534	45	6
**5(0B)**	453	108	19
**6(0B)**	521	61	4
$\bar{x}$	*502.6*	*71.3*	*9.7*

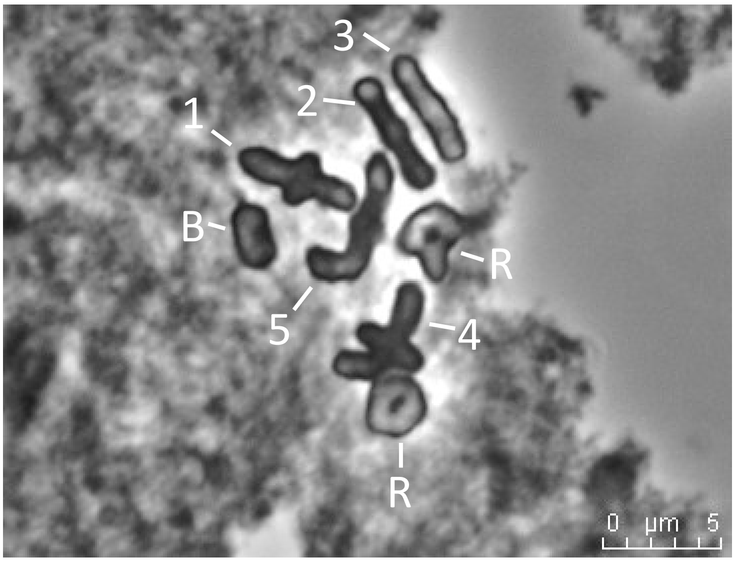

Supplementary Figure S1. Illustration of *L. perenne* chromosome pairing conformations at metaphase. Chiasma positions for rod bivalents are scored as interstitial (1), distal (2,3,5) and proximal (4). R = ring bivalents and B = B chromosome pair.
